# The Role of Urinary NGAL in the Management of Primary Vesicoureteral Reflux in Children

**DOI:** 10.3390/ijms24097904

**Published:** 2023-04-26

**Authors:** Cristina Gavrilovici, Cristian Petru Dusa, Codruta Iliescu Halitchi, Vasile Valeriu Lupu, Elena Lia Spoiala, Roxana Alexandra Bogos, Adriana Mocanu, Mihai Gafencu, Ancuta Lupu, Cristina Stoica, Iuliana Magdalena Starcea

**Affiliations:** 1 Department of Pediatrics, “Grigore T. Popa” University of Medicine and Pharmacy, 700115 Iasi, Romania; 2 Department of Pediatrics, “Victor Babeş” University of Medicine and Pharmacy, 300041 Timisoara, Romania; 3 Department of Pediatrics, “Carol Davila” University of Medicine and Pharmacy, 050474 Bucuresti, Romania

**Keywords:** vesicoureteral reflux, children, NGAL, biomarkers

## Abstract

Vesicoureteral reflux (VUR) is the most frequent congenital urinary tract malformation and an important risk factor for urinary tract infections (UTIs). Up to 50% of children with VUR may develop reflux nephropathy (RN), and the diagnosis and monitoring of renal scars are invasive and costly procedures, so it is paramount to find a non-invasive and accurate method to predict the risk of renal damage. Neutrophil gelatinase-associated lipocalin (NGAL) has already proven to be a good predictive biomarker in acute kidney injuries, but there are few studies that have investigated the role of NGAL in primary VUR in children. Our aim is to review the predictive value of urine NGAL (uNGAL) as a non-invasive biomarker of RN in children with primary VUR, as well as its ability to predict the evolution of chronic kidney disease (CKD). Based on our analysis of the available original studies, uNGAL can be an accurate and reliable biomarker of RN and its progression to CKD. Some studies suggested a good correlation between VUR severity and uNGAL levels, but other studies found no significant correlation. The relationship between VUR severity and uNGAL levels is likely complex and influenced by factors such as UTIs, the timing of the urine sample collection, and the age and overall health of the patient.

## 1. Introduction

Vesicoureteral reflux (VUR) is the most frequent congenital renal malformation and is defined as a backward flow of urine from the bladder into the ureter [1]. It is considered the most important congenital risk factor for the development of urinary tract infections (UTIs) in the pediatric population [2,3]. Primary VUR is considered to be the most common type of congenital anomaly of the kidney and urinary tract (CAKUT) [2,4]. It accounts for up to 44% of cases of CKD in children and is the main cause of end-stage renal disease in children younger than 5 years [5]. Primary VUR occurs in 1% of individuals in a healthy population, 20% of individuals with UTIs, and up to 50% of individuals with recurrent cases of UTIs [6]. Half of children with VUR may develop RN. VUR is asymptomatic and is usually diagnosed during a urinary tract infection [1,2]. VUR is diagnosed in 40% of children presenting febrile UTIs and in 15–20% of children with a history of antenatal hydronephrosis [6]. Consequently, there is a bimodal distribution in the age of presentation of VUR, with one mode occurring at less than 3 months of age and the other at approximately 2–4 years of age [6,7]. There appears to be a gender difference in those affected by VUR; infants with reflux detected during the antenatal period are more likely to be boys, while children with reflux diagnosed following a febrile UTI are more likely to be girls. Additionally, there appears to be a racial difference in those affected with VUR. Caucasian girls are 10 times more likely to have reflux than their African-American counterparts [7].

There are controversies regarding systematic screening for VUR in children with UTIs. According to American Academy of Pediatrics, voiding cystourethrograms (VCUGs) are not routinely recommended in children aged between 2 and 24 months old with a first episode of febrile UTI [8]. There are two reasons: First, they are invasive due to bladder catheterization; in addition, this exposure to the X-rays risks potential urethral damage and iatrogenic infections, and it is not always associated with good parental adherence [9]. Second, there is a lack of effective therapeutic strategies for the lower grades of VUR. On the other hand, those who promote the routine use of VCUGs underline that the potential significant morbidities associated with RN (growth retardation, hypertension, and chronic kidney disease [10] as well as the risk of delayed surgical procedures for high-grade VUR) are important justifications for their position [11].

The routine laboratory tests that are often used as markers for renal injuries include blood urea nitrogen, creatinine, creatinine clearance, and urine sediment, whereas the diagnosis of RN is frequently based on imaging studies such as 99 mTc-dimercaptosuccinic acid (DMSA) renal scintigraphy, an invasive and costly procedure [12].

Thus, urinary biomarkers that are able to predict the association of VUR as well as detect the early progression of renal scars are needed. Therefore, newer, non-invasive, and more precise tools are needed for a better prediction of the risk of kidney damage. Neutrophil gelatinase-associated lipocalin (NGAL) is included in the lipocalin family [13]. NGAL is secreted by the thick limb of the Henle loop and collecting renal ducts [2] and normally accumulates at low levels in different organs (such as the stomach, colon, trachea, kidneys, and lungs) in the healthy population. It increases to high levels in the kidney serum and urine after an ischemic or nephrotoxic injury [1,14].

As NGAL has already proven to be a good predictive biomarker, particularly in acute kidney injuries, our aim is to review its applicability in the early management of primary VUR, where its predictive status is still debated. It is out of our scope to discuss its role in the management of febrile UTIs associated with VUR since NGAL’s importance in acute pyelonephritis has already been proven. Here, we consider only the situations of VUR with no current UTI (at the moment of study enrolment). There have been few studies investigating the role of NGAL only in primary VUR. Taking into account that up to 50% of children with VUR may develop RN and that the diagnosis and monitoring of renal scars is invasive and costly [15,16], it is paramount to find the possibility to use uNGAL for this purpose.

Thus, our aim is to review the predictive value of this non-invasive biomarker for RN in children with primary VUR as well as its ability to predict the evolution of CKD in these children. Chronic kidney disease (CKD) in children contributes to an additional burden on the global health system. The use of new biomarkers to predict disease onset and progression has grown tremendously over the past decade. The discovery of biomarkers offers perspectives for the early anticipation of renal degradation towards the advanced stages of CKD, thus contributing to decreasing the rate of disease progression.

We performed an electronic search in the PubMed and Embase databases using the following search terms: primary vesicoureteral reflux AND NGAL OR (Neutrophil Gelatinase-associated Lipocalin)) AND children (OR pediatric OR paediatric). The databases were searched from the date of inception until March 2023. Articles were screened by titles and abstracts. Full texts of relevant articles were retrieved and independently assessed. We limited our search to the English language, humans, and original studies. Review articles, case reports, and letters were excluded. All of the significant articles were manually searched to further identify any additional eligible studies. Among these, it was evident that uNGAL was used with two main purposes: (1) as a diagnostic tool in children with primary VUR and (2) as a predictive tool for RN.

Eight original articles were included for a deep analysis in this review. A summary of the main results of these studies is presented in Table 1. Another 58 selected articles were used to discuss the topic.

## 2. Discussion

Both plasma NGAL (pNGAL) and uNGAL are markers of kidney injury. While raised levels of pNGAL are indicative of systemic inflammation, significant increases in uNGAL are seen in conditions involving the urinary tract [5] Nishida et al. [24] also demonstrated the superiority of uNGAL over pNGAL as a biomarker of the progression to chronic kidney diseases. The etiological factors recognized in CKD in children are dominated by congenital abnormalities of the kidney and urinary tract (CAKUT) and hereditary nephropathies, which are responsible for two thirds of all cases of CKD in developed countries, as shown by current reports from the United States [25], Italy [26], Belgium [27], France [28], and Sweden [29]. A reduction in the number of nephrons associated with intrauterine growth restriction and pediatric obesity are additional risk factors for the evolution of CKD in children. Uwaezuoke reported the role of biomarkers as predictors of the onset of CKD and the risk of progression, such as in nephrotic syndrome, chronic pyelonephritis, congenital obstructive nephropathy, and diabetic nephropathy [30].

### 2.1. Primary Vesicoureteral Reflux in Children—Focus on Genetics

VUR is the most common congenital anomaly of the kidney and urinary tract and is a major risk factor for pyelonephritic scars and CKD in children. The global incidence of VUR is estimated to be 10% in the general population. Since the 2000s, Feather has published the results of the first genome-wide search for VUR and RN using the GENEHUNTER program. VUR maps to a locus on chromosome 1p13 and 2q37 under autosomal dominant inheritance [31]. VUR has been described with a prevalence of 27–51% in siblings of patients with VUR, and a 66% rate of VUR has been observed in children whose parents had reflux [32]. However, it is known that VUR can resolve spontaneously in the first 3 years of life; therefore, the exact prevalence in family members could be underestimated.

### 2.2. Neutrophil Gelatinase-Associated Lipocalin—Current Knowledge

NGAL is a 25 kDa protein belonging to the lipocalin superfamily. Initially found in activated neutrophils, it can be produced in kidney tubular cells in response to various injuries. uNGAL has been proposed to be an early predictor of acute kidney injury (AKI). Both pNGAL and uNGAL have been studied in relation to kidney injury. Nickolas et al. reported the first study of urinary NGAL in adults admitted to emergency departments and demonstrated that uNGAL has a good predictive capability for intrinsic AKI. This study also suggested that NGAL could be useful as a predictive marker for adverse clinical outcomes such as the need for dialysis and admission to the ICU [33]. Shapiro et al. determined that a level of plasma NGAL above 150 ng/mL at the moment of presentation is predictive of AKI occurrence within 3 days of hospitalization. There is a distinct difference between uNGAL, which is specific to injured epithelial cells of the distal nephron, and plasma NGAL, which results from tubular reabsorption from the injured kidney as well as from organs that ultimately crosstalk with the kidney [34]. NGAL was tested in multiple renal diseases, starting with AKI, and multiple causes of chronic kidney disease, such as nephrotic syndrome, type 1 diabetes, and urinary tract malformations, including VUR. After the first study by Mishra [35], Parikh [36], Krawczeski [37], and Bennett [38] also confirmed positive predictive values of uNGAL or pNGAL for AKI after cardiac surgery in pediatric or neonatal contexts. Urinary tract infections are one of the trademarks of subjacent malformations. In this sense, the role of NGAL in pediatric UTIs had to be determined. As a diagnostic tool, Shaikh et al. found that uNGAL was more sensitive and specific for a positive UTI diagnosis than leukocyte esterase [39]. Moreover, Jagadesan found that uNGAL was more specific than pyuria [40], while Kim Byunh Kwan [41] and Ji Hym Moon [42] found it to be more specific than C-reactive protein for the diagnosis of UTIs. Some studies suggested that NGAL could be used to differentiate between acute pyelonephritis and lower UTI, with plasma NGAL having better results [43,44,45]. Other causes of CKD include nephrotic syndrome and type 1 diabetes. It has been observed that uNGAL may serve as a differentiation marker between minimal-change nephrotic syndrome and focal and segmental glomerulosclerosis [46]. Renal survival has been directly associated with proteinuria control in long-term cohort studies in patients with GSFS, so a marker to predict the damage to renal function in glomerular nephropathy can be very useful. uNGAL may also be useful as an early diagnostic marker of diabetic nephropathy in type 1 diabetes patients that is superior to microalbuminuria [47]. By contrast, some malformations are not prone to UTIs or recurrent UTIs. They do not present with the edema of nephrotic syndrome or symptoms related to diabetes. They may not present as modifications on an ultrasound [48]. VUR represents a true diagnostic challenge.

### 2.3. The Diagnostic Value of uNGAL in Children with Primary VUR

Depending on the severity of a kidney injury, increased NGAL production and decreased NGAL reabsorption may occur in patients with renal tubular damage. uNGAL has already proven its validity as a biomarker for the diagnosis of children with acute UTI and for steroid responses in idiopathic nephrotic syndrome or in children with different urologic conditions [17,18]. Nickavar et al. [19] recognized the diagnostic role of uNGAL in patients with primary VUR and demonstrated in a prospective case control study of 69 small children (2–3 years old) the accuracy of uNGAL/uCr for VUR diagnosis, demonstrating high specificity, sensitivity, and accuracy. Therefore, imaging exploration (e.g., scintigraphy) might not be compulsory for VUR management in children with low uNGAL. However, there was no correlation between uNGAL and the severity of VUR. In a recent study, Amiri et al. [2] also found higher levels of uNGAL in VUR patients vs. control and higher but not significant levels of uNGAL in the severe compared to mild/moderate forms of VUR as well as in bilateral vs. unilateral involvement. However, when adjusting for uCr, significant differences in the uNGAL/uCr ratio between study groups (patients and controls) and between the patient subgroups, according to the severity of the disease, have been found. No significant differences were found for uNGAL and the uNGAL/uCr ratios between the patients with and without renal scars. This means that uNGAL could not predict RN and could only predict the severity of VUR. In a similar sample (71 children aged 1–5 years), Eskandarifar et al. [20] confirmed significant differences between the uNGAL levels and the uNGAL/uCr ratios in the VUR group compared with a healthy group. Furthermore, contrary to Nickavar [19] and Amiri [2], they detected a significant correlation between uNGAL and the VUR grade. Thus, these authors, referring specifically to the diagnostic role of NGAL in VUR (with no attempt to describe the predictive value for renal scars), reinforced that VCUGs should not be routinely performed in small children after a first febrile UTI, as long as the NGAL/Cr ratio can help, at least in the preliminary stages of VUR management. As there are disagreements regarding the relation to the severity of VUR, the role of uNGAL in predicting the severity of reflux is not well established.

### 2.4. NGAL as a Predictive Tool for Renal Scarring and RN

Four original studies aimed specifically to assess the predictive value of uNGAL for the development of RN after they had already demonstrated the significant relationship between increased uNGAL and VUR [4,21,22,23]. Apart from this, the correlation with the severity of VUR as well as a comparison with other biomarkers such as kidney injury molecule-1 (KIM 1) and liver-type fatty-acid-binding protein (L-FABP) was also sought. The Parmaksız et al. study [1] had the most complex study design. They studied uNGAL in a sample of 123 children with primary VUR. They split the sample into four groups: group A—VUR + renal scars, group B—VUR without renal scars, group C—renal scars and resolved VUR, group D—resolved VUR + no renal scars, and group E—healthy children. The uNGAL/uCr ratio was significantly higher in the groups with scarring than in those without it, demonstrating that uNGAL corelates with scarring and not with VUR (contrary to Amiri [2]). Moreover, the uNGAL levels in patients with renal scars and VUR (group A) were significantly higher than in the group with VUR without renal scars (group B), the group with fully resolved VUR without renal scars (group D), and the control group, demonstrating that renal scars were corelated with increased uNGAL levels. A particularity of this study was that it also demonstrated a correlation with renal scars: the uNGAL/uCr ratio significantly increased with the severity of renal scars. Similar outcomes were found in Ichino’ study [18] aiming to assess the predictive value of uNGAL for renal scar development in children with primary VUR. Their results demonstrated a significant relationship between the presence of renal scars and high uNGAL values. However, no significant relationship between uNGAL and the severity of VUR has been proven. Thus, contrary to the results reported by Amiri [2] and Eskandarifar [20], in the studies by Ichino [18], Nickawar [19], and Parmaksız [21], the uNGAL levels did not appear to reflect the severity of VUR. Parmaksız’s results [1] were consistent with Naik’s conclusions: the uNGAL values were higher in children with renal scars than in those without renal scars [22]. Unlike uNGAL, uKIM-1 and uKIM-1/Cr were not able to predict renal scar formation. Anand et al. [4] underlined the importance of NGAL in predicting the functional deterioration associated with VUR. They studied uNGAL (among three other biomarkers: trefoil family factors (TFF 1 and 3) and microalbuminuria), in a sample of 50 children with congenital anomalies of the kidney and urinary tract (of which 18 had primary VUR). Their main outcome was a progressive decline in renal function (a decrease in GFR from ≥60 to <60 mL/min/1.73 m^2^ and/or new-onset kidney scars or the growth of previous scars on DMSA scans. uNGAL proved to be an accurate biomarker for the progression of chronic kidney disease: the median concentrations of NGAL were significantly higher in children with the progressive deterioration of kidney function. In the newer study by Eskandarifar, the average urinary NGAL level was found to be 524.05 ± 166.65 ng/dL in the group of patients with renal scars, while in the group without renal scars, it was 125.77 ± 61.06 ng/dL (*p* < 0.05). The authors showed that urinary NGAL levels had a positive predictive value of 100% and a negative predictive value of 81.25% for predicting the presence of renal scars [23]. In the study by Eskandarifar, the patients with renal scars had significantly higher levels of NGAL in their urine compared to those without renal scars, similar to the study by Parmaksız [20,21]. In a mouse model, Han et al. reported that there was a significant increase in NGAL levels two weeks after pyelonephritis that did not definitively return to basal levels after 4–6 weeks. The initial increase in NGAL levels was the result of an acute inflammatory response, but the persistent levels after six weeks indicated that the origin of the high NGAL levels was related to tubular dysfunction [48].

### 2.5. NGAL—Predictive Tool for CKD Progression in Children

Chronic kidney disease (CKD) involves irreversible structural and/or functional damage to the kidneys, with evolutionary potential and an evolution longer than 3 months. It is usually accompanied by albuminuria and histopathological and imaging changes characteristic of its etiological type [49]. A drop in the glomerular filtration rate below 60 mL/min/1.73 m^2^ is considered to be a sign of advanced CKD [50]. Due to the ever-increasing number of patients with CKD, the disease is called “the epidemic of the 21st century”. The incidence of CKD in children under age 16 is 1.5–3 per 1,000,000 [51]. The early stages of CKD are asymptomatic most of the time, so the diagnosis can be delayed in many cases. It is known, however, that the early diagnosis of CKD allows the implementation of an effective strategy to slow down the progression of the disease [52]. Currently, the traditional markers used for the diagnosis of CKD, i.e., creatinine, urea, GFR, albuminuria, and proteinuria, do not have a high sensitivity [53]. GFR reflects the total number of functional nephrons. Although measuring GFR using inulin clearance is still the gold standard, the method is far too laborious and is not used in clinical practice. The Schwartz formula, which was developed in 1976, uses serum creatinine (mg/dL, measured using the Jaffe test), height (cm), and the coefficient k, which is proportional to muscle mass and depends on age and sex. However, the error of GFR calculated from serum creatinine has been shown to be approximately ±20% and ±30–40% in children [54]. In children under 2 years of age, GFR can also be underestimated due to the immaturity of the urinary tract. The measurement of endogenous creatinine also has its limitations. The creatinine level depends, among others, on muscle mass and diet, which can generate significant variation in children, especially in children under 3 years old. Although albuminuria and proteinuria are important parameters for the assessment of the CKD status in adults, they are not always applicable in pediatric practice. In accordance with the 2012 KDIGO guideline [55], the range of values for albuminuria in children is the same as in adults, but these criteria are not useful in children under 2 years of age due to renal immaturity and protein reabsorption in the proximal convoluted tubule compared to adults [56]. In addition, CKD in children is often due to congenital abnormalities associated with the tubular loss of albumin; therefore, albuminuria is a less sensitive renal marker in this category of patients [57]. Due to the mentioned limitations, there is a continuous search for new, early, sensitive, and specific markers of kidney damage, whose introduction into daily clinical practice would lead to the early establishment of the diagnosis of CKD. New markers with particular potential in the diagnosis of CKD include uromodulin, KIM-1, fibroblast growth factor 23 (FGF23), urinary N-acetyl-β-D-glucosaminidase (NAG), NGAL, glomerular biomarker soluble urokinase-type plasminogen activator receptor (suPAR), and urinary retinol-binding protein 4 (RBP4). Serum creatinine, blood urea nitrogen, creatinine clearance, urinalysis, and radiological findings have traditionally been the markers used to indicate renal damage in VUR. However, these markers lack the sensitivity or specificity to accurately diagnose kidney damage and scarring. On the other hand, a high level of urinary NGAL has been associated with kidney damage and an increased risk of kidney scarring, making it a potentially useful diagnostic marker [23]. Bolignano et al. evaluated the utility of NGAL as an independent marker of CKD progression. The serum and urinary levels of NGAL were inversely proportional to the decrease in GFR [56]. Because NGAL measurements were more reliable in patients with stage-1–3 CKD than in patients in stages 4 or 5, NGAL was considered to be a useful marker for the diagnosis of early CKD. In the CRISIS study, Alderson et al. also found NGAL to be a marker of CKD [58]. In addition, there appears to be a correlation between NGAL and serum creatinine levels. Mori et al. [59] postulated in the “Forest Fire Theory” that increasing the concentration of NGAL is not only a consequence of decreased renal clearance in CKD patients but also the result of increased production of this protein in damaged tubular epithelial cells. According to this theory, the NGAL concentration is assumed to reflect the activity of ongoing processes of kidney damage during CKD. While the increase in the serum creatinine concentration is the result of the loss of a number of functional nephrons, the increase in NGAL reflects the rate of progression of CKD [60].

### 2.6. Disadvantages and Limitations in Using uNGAL in Renal Pathology in Children

Most research has focused on the diagnostic value of NGAL for AKI. However, this biomarker has been evaluated in a variety of renal diseases, such as VUR, glomerulopathies, polycystic kidney disease, and CKD. The progression of kidney disease can be effectively slowed if a kidney disease or injury (e.g., AKI) is diagnosed early. The use of non-invasive biomarkers can effectively estimate renal lesions with evolutionary potential, especially in communities with a high prevalence of CKD in children. However, in a study by De Silva [61] with an emphasis on the roles of uNGAL and KIM-1 in renal injury, the authors concluded that the concentrations of the biomarkers may vary depending on several factors, including the water intake, time of sample collection, hydration status, and urine output. Hence, biomarker levels adjusted for creatinine may be more reliable. Another study by Bellos et al. [60] discussed the disadvantages of the determination of uNGAL in small children. As small kidneys lack sufficient renal tubular cells with the residual regenerative capacity to produce high levels of NGAL, lower specificity and sensitivity findings were detected in small children [21]. Urinary NGAL is not typically used as a screening tool for detecting renal scars caused by VUR due to its low sensitivity. However, it can be a useful diagnostic marker for identifying renal scars, as it has a high specificity, as demonstrated by Eskandarifar et al. in their last study [23]. Individual biological variations in urinary NGAL have a wide range of values. However, more attention should be paid to the biological variations in both urinary and systemic NGAL, as these studies only tested the biological variation in healthy individuals, not in patients with renal injuries. In addition, some reagents have not been validated by clinical studies, so their analytical performances may be questionable. These practical issues pose great challenges for the standardization of NGAL testing [62]. These results illustrate that urinary NGAL is a useful biomarker for risk classification and the prediction of clinical outcomes in CKD patients. However, due to the various etiologies of CKD, close attention must be paid to the usefulness of NGAL in different kinds of CKD. For example, children with CKD due to neurogenic bladder and secondary VUR that were dependent on wheelchairs had higher UNGAL levels than those on foot. According to Bagińska’s results, the patient’s level of mobility is another factor affecting uNGAL [63]. This finding suggests that children who are wheelchair-dependent have a higher risk of tubular dysfunction than others. The most likely explanation for this result is the fact that the patients are wheelchair-dependent and that the precise estimation of the renal function with the available classical parameters (serum creatinine) could generate erroneous results [64]. In this context, uNGAL may be the promising marker of renal function in this patient population. We tried to summarize the advantages and limitations of using uNGAL in primary VUR in children in Table 2.

The next years of cost-effectiveness research in the use of biomarkers in the early diagnosis of kidney diseases are likely decisive. There are already studies evaluating the usefulness of new biomarkers since 2012, when KDIGO recommended them for the early diagnosis, differential diagnosis, and prognosis of kidney diseases, especially AKI. As such, more detailed models that closely reproduce the progression of AKI and/or CKD would greatly help to evaluate the profitability of new diagnostic and surveillance technologies in the field of renal pathology.

## 3. Conclusions

There have been some studies dedicated to the role of uNGAL in primary VUR management in children, outside the association with a concomitant UTI. From our analysis of the available original studies, there is no doubt that uNGAL is an accurate and reliable biomarker, not only for UTIs but also for primary VUR. It also shows a good predictive ability for RN and its progression to CKD, as it was proven to be associated with renal scars. The severity of VUR was not clearly demonstrated to be linked with increased uNGAL, as the results were contradictory. uNGAL is better than uKIM in predicting renal scarring when assessed alone. The NGAL concentration reflects the progressive activity of kidney damage during CKD. In several studies, by demonstrating a correlation between uNGAL and VUR, irrespective of the severity of the reflux, it was suggested that the monitoring of uNGAL will help to establish the opportunity for surgical management. Consequently, imaging studies might become unnecessary for the diagnosis of VUR in children with low uNGAL excretion. uNGAL/uCr is superior to other biomarkers such as uKIM-1/uCr in predicting renal scars and VUR. However, additional multi-center studies in larger pediatric populations are needed in order to confirm the potential application of uNGAL for the diagnosis and management of patients with VUR and kidney damage.

## Figures and Tables

**Table 1:** Studies depicting the role of NGAL in VUR management in children.

Authors, Year of Publication	Study Design and Study Population	Aims	Sample Size and Age	Main Results	Limitation of uNGAL Determination	Conclusions
**1.** **Amiri et al., 2020 [17]**	Prospective case-control study	uNGAL in primary VUR	- 63 children with primary VUR - 72 healthy controls - 2 months–12 years old	- no significant difference in uNGAL between mild/moderate and severe VUR - significant differences in uNGAL/uCr between study groups	- none	- higher uNGAL/uCr ratio in severe vs. mild/moderate VUR
**2.** **Ichino et al., 2010 [18]**	Prospective case-control study	uNGAL—biomarker of renal scars in VUR	- 34 children with primary VUR - 28 healthy controls - 5 months–11 years old	- significantly higher uNGAL in the VUR group - no significant differences in uNGAL between the lower and higher grades of VUR - significantly higher uNGAL in patients with renal scars	- no correlation with VUR grade	- uNGAL—non-invasive diagnostic and predictive biomarker for renal scars - uNGAL is biologically stable and resistant to protein degradation. The parents collected urine samples from small children (less than 3 years) at home for transport to the hospital.
**3.** **Nickavar et al., 2020 [19]**	Prospective case-control study	uNGAL in primary VUR	- 32 children with primary VUR - 37 children without VUR - 36.84 ± 28.16 months for children with VUR - 32.32 ± 29.08 months for children without VUR	- significantly higher uNGAL and uNGAL/uCr ratio in patients with VUR - significantly higher uNGAL in patients with renal scars	- none	- uNGAL/uCr ratio—good accuracy, high specificity, and high sensitivity as a non-invasive biomarker of primary VUR - Children with decreased uNGAL did not need further imaging studies.
**4.** **Eskandarifar et al., 2021 [20]**	Prospective cohort study	uNGAL in primary VUR	- 34 children with VUR - 37 children without VUR - 1–5 years old	- significantly higher uNGAL and uNGAL/uCr ratio in patients with VUR	- uNGAL cannot replace VCUG, the gold standard for VUR diagnosis.	- uNGAL and - uNGAL/Cr ratio—biomarkers for renal scars in VUR patients
**5.** **Parmaksız et al., 2016 [21]**	Prospective case-control study Patients with VUR were divided as follows: A: VUR with renal scarring; B: VUR without renal scarring; C: renal scarring and remitted VUR; D: without renal scarring or remitted VUR; E healthy patients.	uNGAL, KIM-1, and L-FABP in RN	- 123 children with primary VUR - 30 healthy controls - Group A: 8.3 ± 2.6 years old - Group B: 9.2 ± 3.2 years old - Group C: 9.7 ± 3.2 years old - Group D: 10.6 ± 2.9 years old - Group E: 9.5 ± 2.9 years old	- significantly higher uNGAL and uNGAL/Cr ratio in patients with renal scars - KIM-1/uCr ratio similar in all five study groups - no significant differences in KIM-1 or L-FABP between mild/moderate and severe VUR	- The kidneys of small children have the capacity to produce high levels of NGAL. - There was lower specificity and sensitivity in children.	- uNGAL—non-invasive predictive biomarker for renal scars in RN - uNGAL is more sensitive than uKIM-1 and uL-FABP in predicting renal scars.
**6.** **Naik et al., 2022 [22]**	Cross-sectional observational study	uNGAL and KIM-1 in VUR and renal scars	- 94 patients with VUR - 0–16 years old	- significantly higher uNGAL in patients with renal scars - low prognostic value of uKIM-1 and uKIM-1/uCr in patients with renal scars	- none	uNGAL predicts renal scars in primary VUR.
**7.** **Anand et al., 2021 [4]**	Prospective case control study	TFFs, uNGAL, and microalbuminuria in CAKUT patients	- 18 children with VUR - 20 age-matched healthy controls - 0–14 years old	- significantly higher TFFs, uNGAL, and microalbumin in patients with CAKUT	- none	uNGAL—the strongest predictor of functional deterioration in RN
**8.** **Eskandarifar et al., 2023 [23]**	Prospective cohort study	uNGAL in VUR and renal scars	- 92 children with VUR (grades 2 to 5) - 40 with renal scars - 52 without renal scars - 3–60 months old	- significantly higher uNGAL in patients with renal scars	- not a good test for screening or early diagnosis due to its low sensitivity	uNGAL—strong predictor of renal scars in primary VUR

VCUG—voiding cystourethrograms; VUR—vesicoureteral reflux; UTI—urinary tract infection; CKD—chronic kidney disease; uNGAL—urine neutrophil gelatinase-associated lipocalin; uCr—urinary creatinine; uNGAL/uCr ratio—urine neutrophil gelatinase-associated lipocalin/urinary creatinine ratio; TFFs—trefoil family factor, small children (less than 3 years); uL-FABP—liver-type fatty-acid-binding protein; uKIM-1—urinary kidney injury molecule 1; CAKUT—congenital anomalies of kidney and urinary tract; KIM-1/uCr ratio—urinary kidney injury molecule 1/urinary creatinine ratio.

**Table 2:** The advantages and limitations of the assay of uNGAL in primary VUR in children.

	The Advantages	The Limitations
uNGAL	- biologically stable, resistant to degradation, and useful for collecting urine samples from small children - non-invasive predictive biomarker for renal scars in RN - more sensitive than uKIM-1 and uL-FABP in predicting renal scars - the strongest predictor of the functional deterioration of the kidney in RN - useful marker for the diagnosis of early CKD (stages 1–3) - uNGAL/uCr ratio—better accuracy, high specificity, and high sensitivity compared to uNGAL alone in primary VUR - Further imaging studies are not necessary if uNGAL is decreased.	- cannot replace VCUG for VUR diagnosis - Small kidneys lack sufficient renal tubular cells with the residual regenerative capacity to produce high levels of NGAL, so specificity and sensitivity are lower in small children. - a wide range of values due to individual biological variations - no significant differences in uNGAL between the lower and higher grades of VUR - the high cost

VCUG—voiding cystourethrograms; VUR—vesicoureteral reflux; CKD—chronic kidney disease; uNGAL—urine neutrophil gelatinase-associated lipocalin; uNGAL/uCr ratio—urine neutrophil gelatinase-associated lipocalin/urinary creatinine ratio; uL-FABP—liver-type fatty-acid-binding protein; uKIM-1—urinary kidney injury molecule 1.
